# Supplementary material for: A unicellular cyanobacterium relies on sodium energetics to fix N2
Source: Nat Commun. 2024 Nov 9;15:9716. doi: 10.1038/s41467-024-53978-9 (PMC11550448; doi:10.1038/s41467-024-53978-9)
Supplement: Supplementary file 1 — Supplementary Information [file 41467_2024_53978_MOESM1_ESM.pdf]

Supplementary information for:

A unicellular cyanobacterium relies on sodium energetics to fix N_2

Si Tang[1], Xueyu Cheng[1], Yaqing Liu[1], Lu Liu[1], Dai Liu[1], Qi Yan[1], Jianming Zhu[1], Jin Zhou[1], Yuyang Jiang[2,3], Katrin Hammerschmidt[4,*], Zhonghua Cai[1,2,5,*]

[1] Shenzhen Public Platform for Screening and Application of Marine Microbial Resources, Tsinghua Shenzhen International Graduate School, Shenzhen, 518055, Guangdong Province, PR China.

[2] National Innovation Center for Molecular Drug, Shenzhen, 518055, Guangdong Province, PR China.

[3] School of Pharmaceutical Sciences, Tsinghua University, Beijing, 100084, PR China.

[4] Institute of General Microbiology, Kiel University, Kiel, Germany.

[5] Technology Innovation Center for Marine Ecology and Human Factor Assessment of Natural Resources Ministry, Tsinghua Shenzhen International Graduate School, Shenzhen, 518055, Guangdong Province, PR China.

* Correspondence:

katrinhammerschmidt@googlemail.com (K.H.), caizh@sz.tsinghua.edu.cn (ZH.C.)

Includes:

Supplementary figures 1-8

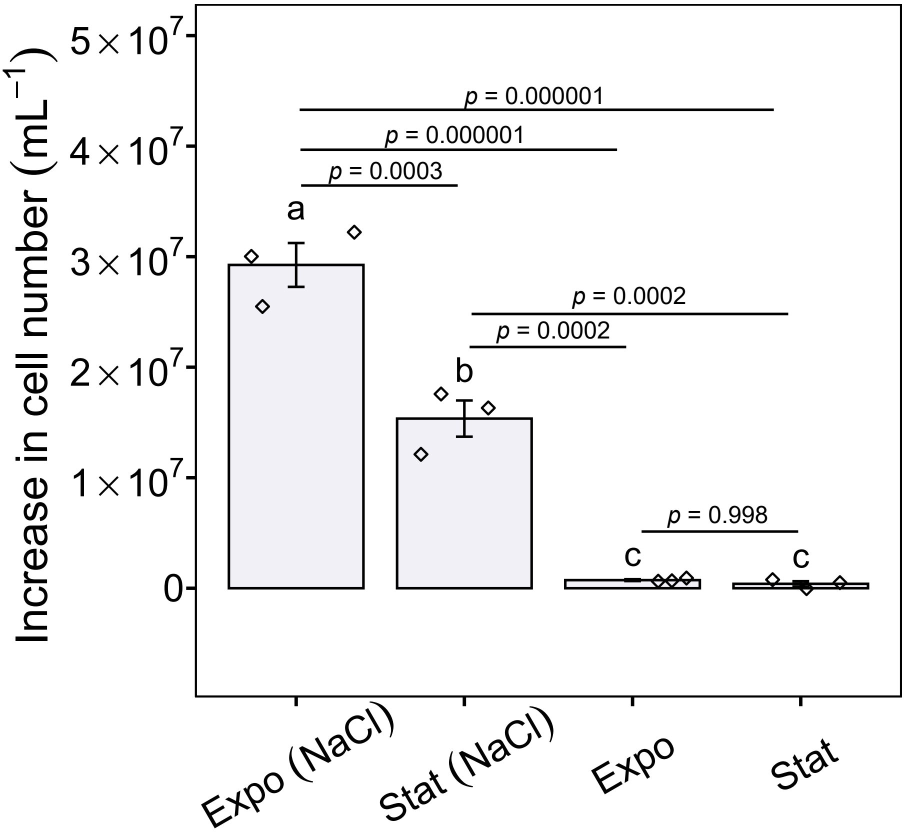

Supplementary Fig. 1. Population growth over seven days from different growth stages under N deprivation. Cells were grown in $BG11_0$ with or without NaCl (18 g/L). Expo (NaCl), exponential phase cells with NaCl; Stat (NaCl), stationary phase cells with NaCl; Expo, exponential phase cells without NaCl; Stat, stationary phase cells without NaCl. Exponential phase cells refer to cultures 7 days post inoculation, whereas stationary phase refers to 20-day-old cultures post inoculation. Notably, N_2-fixing stationary phase cells grew significantly slower than exponential phase cells in the presence of NaCl. This is because stationary phase cells require more time to recover growth and therefore have a longer lag time. The graph shows the mean ± standard deviation (n = 3 biologically independent samples). Different letters on each bar indicate statistical significance ($p < 0.05$) calculated by one-way ANOVA with Tukey's HSD post-hoc analysis over all populations tested. Source data are provided as a Source Data file.

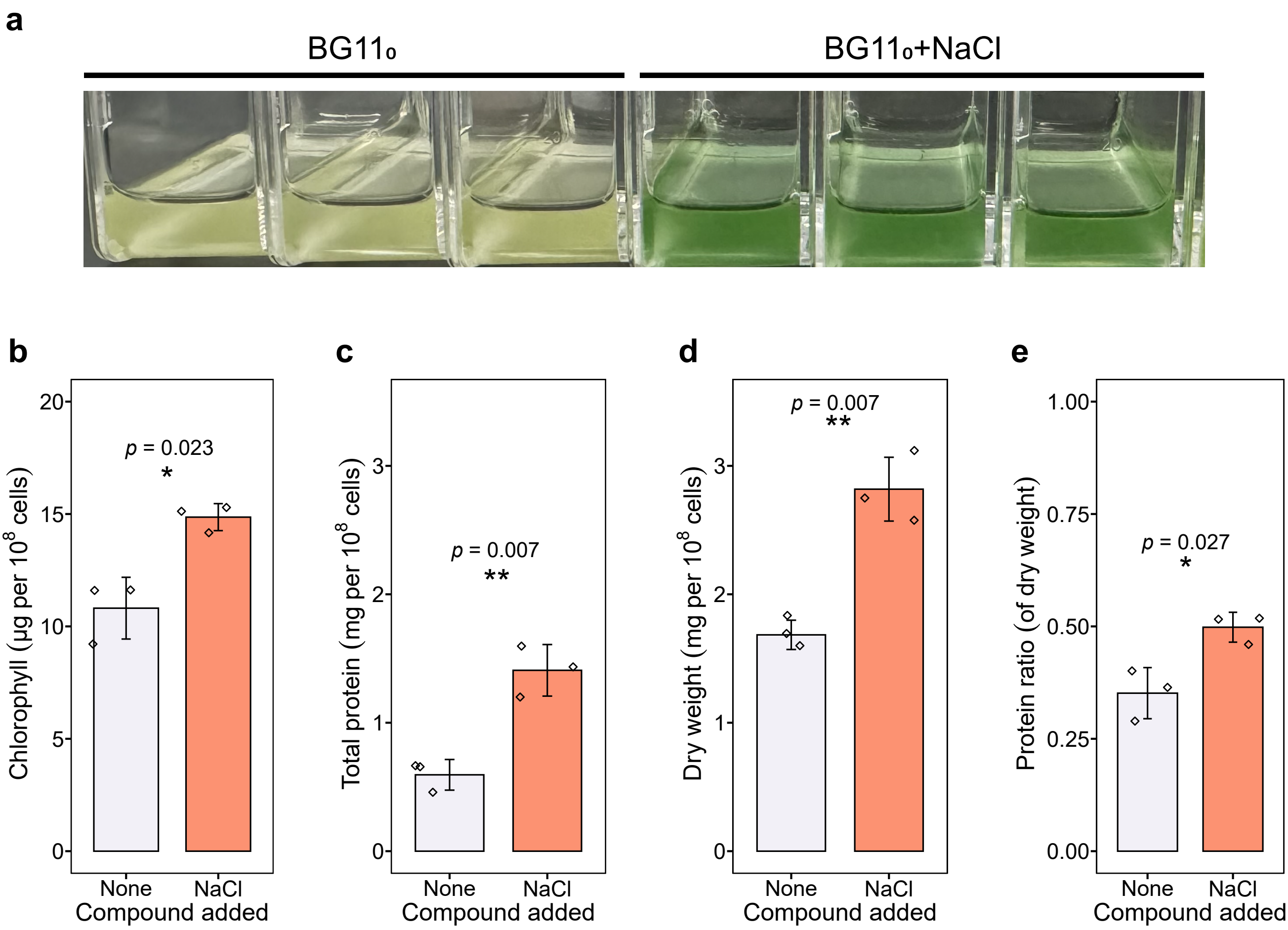

Supplementary Fig. 2. Images and physiological parameters of cells in the absence or presence of NaCl under diazotrophy. a Images of cells (day 7) grown in $BG11_0$ or $BG11_0$ with NaCl (18 g/L) ($BG11_0$ (NaCl)). Similar results were observed in six independent experiments. **b** Chlorophyll content. **c** Total protein. **d** Dry weight. **e** Protein to dry weight ratio. “None” indicates that no additional substances were added to $BG11_0$ while “NaCl” denotes the addition of additional NaCl (18 g/L) to $BG11_0$. The graphs show the mean ± standard deviation (n = 3 biologically independent samples). Corresponding significance markers represent statistical significance calculated by two-tailed Welch’s *t* test. Significance: ns (no significance), *($p < 0.05$), **($p < 0.01$), ***($p < 0.001$), ****($p < 0.0001$). Source data are provided as a Source Data file.

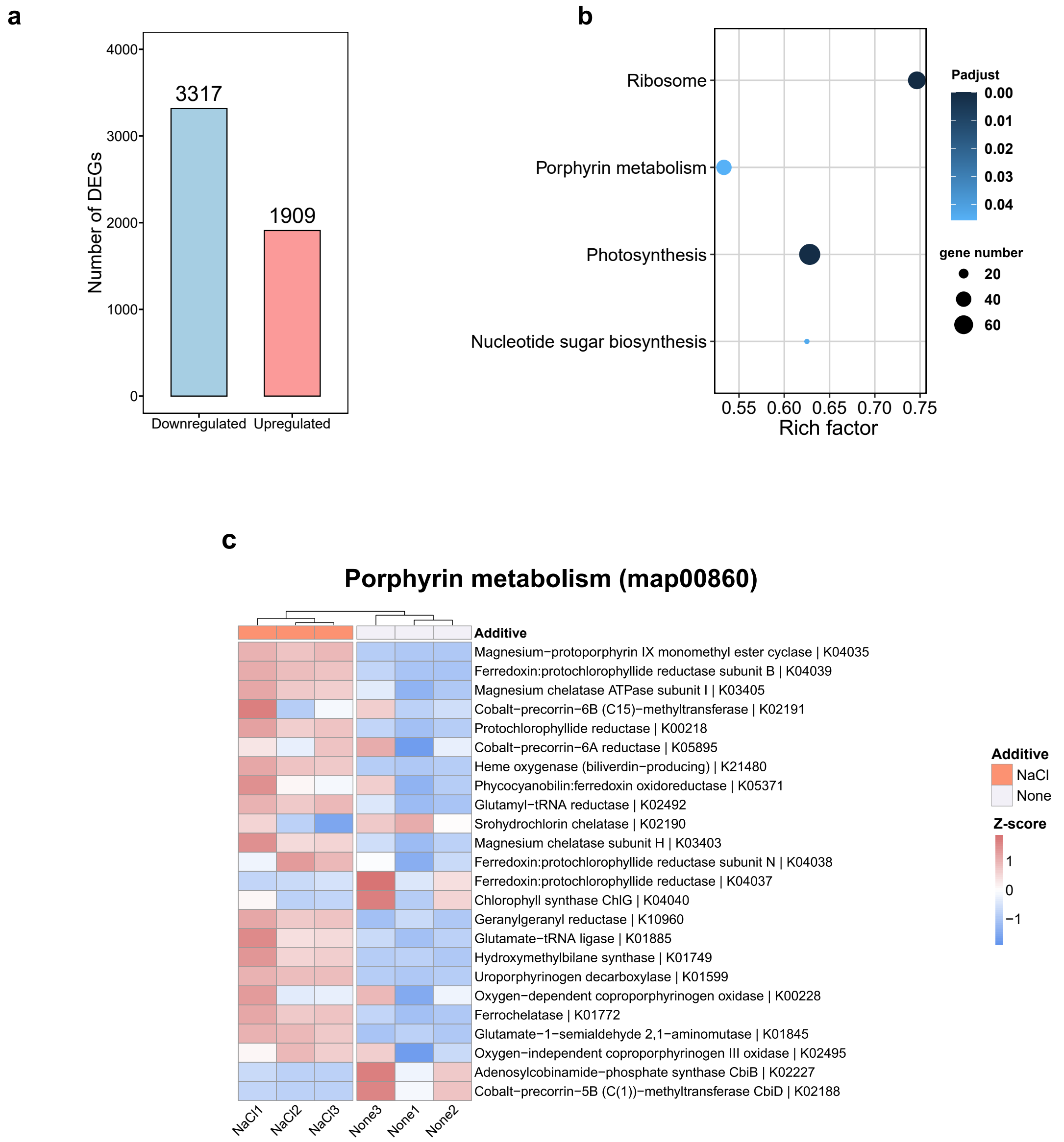

Supplementary Fig. 3. Transcriptomes and key functional enrichment assay of DEGs. a Number of significantly up- or downregulated DEGs (number of genes denoted at the top). **b** Analysis of enriched KEGG Pathways containing DEGs with a Padjust value < 0.05. The size of the dot reflects the number of genes for each pathway, and the colour reflects the *p* value. **c** Heatmap analysis showing the DEGs enriched for porphyrin metabolism (map00860). Column dendrograms show similarity based on Euclidean distance and hierarchical clustering. Gene clusters were determined by k-means clustering with Euclidean distance. KEGG annotations were assigned from the genome annotation. The heatmap colour gradient shows low gene expression (blue) and high gene expression (red). "NaCl" denotes the addition of NaCl (18 g/L) to $BG11_0$, while "None" indicates that no additional substances were added to $BG11_0$. Source data are provided as a Source Data file.

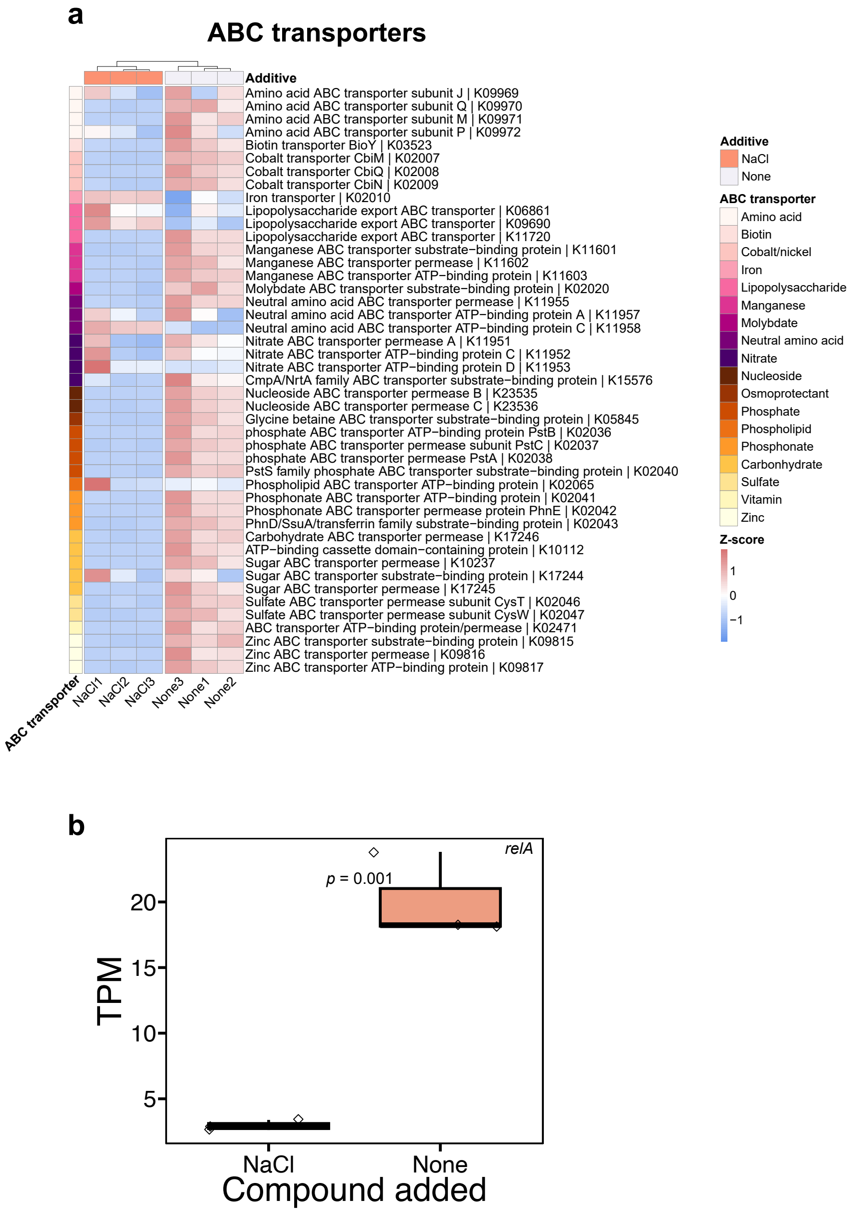
a
ABC transporters
Additive
Amino acid ABC transporter subunit J | K09969
Amino acid ABC transporter subunit Q | K09970
Amino acid ABC transporter subunit M | K09971
Amino acid ABC transporter subunit P | K09972
Biotin transporter BioY | K03523
Cobalt transporter CbiM | K02007
Cobalt transporter CbiQ | K02008
Cobalt transporter CbiN | K02009
Iron transporter | K02010
Lipopolysaccharide export ABC transporter | K06861
Lipopolysaccharide export ABC transporter | K09690
Lipopolysaccharide export ABC transporter | K11720
Manganese ABC transporter substrate−binding protein | K11601
Manganese ABC transporter permease | K11602
Manganese ABC transporter ATP−binding protein | K11603
Molybdate ABC transporter substrate−binding protein | K02020
Neutral amino acid ABC transporter permease | K11955
Neutral amino acid ABC transporter ATP−binding protein A | K11957
Neutral amino acid ABC transporter ATP−binding protein C | K11958
Nitrate ABC transporter permease A | K11951
Nitrate ABC transporter ATP−binding protein C | K11952
Nitrate ABC transporter ATP−binding protein D | K11953
CmpA/NrtA family ABC transporter substrate−binding protein | K15576
Nucleoside ABC transporter permease B | K23535
Nucleoside ABC transporter permease C | K23536
Glycine betaine ABC transporter substrate−binding protein | K05845
phosphate ABC transporter ATP−binding protein PstB | K02036
phosphate ABC transporter permease subunit PstC | K02037
phosphate ABC transporter permease PstA | K02038
PstS family phosphate ABC transporter substrate−binding protein | K02040
Phospholipid ABC transporter ATP−binding protein | K02065
Phosphonate ABC transporter ATP−binding protein | K02041
Phosphonate ABC transporter permease protein PhnE | K02042
PhnD/SsuA/transferrin family substrate−binding protein | K02043
Carbohydrate ABC transporter permease | K17246
ATP−binding cassette domain−containing protein | K10112
Sugar ABC transporter permease | K10237
Sugar ABC transporter substrate−binding protein | K17244
Sugar ABC transporter permease | K17245
Sulfate ABC transporter permease subunit CysT | K02046
Sulfate ABC transporter permease subunit CysW | K02047
ABC transporter ATP−binding protein/permease | K02471
Zinc ABC transporter substrate−binding protein | K09815
Zinc ABC transporter permease | K09816
Zinc ABC transporter ATP−binding protein | K09817
ABC transporter
NaCl1
NaCl2
NaCl3
None3
None1
None2
Additive
NaCl
None
ABC transporter
Amino acid
Biotin
Cobalt/nickel
Iron
Lipopolysaccharide
Manganese
Molybdate
Neutral amino acid
Nitrate
Nucleoside
Osmoprotectant
Phosphate
Phospholipid
Phosphonate
Carbonhydrate
Sulfate
Vitamin
Zinc
Z-score
1
0
−1
b
relA
p = 0.001
TPM
20
15
10
5
NaCl
None
Compound added

Supplementary Fig. 4. Enrichment analysis of ABC transporters and transcript analysis of the stringent response signal-encoding gene *relA*. a Heatmap analysis showing the DEGs enriched for nutrient transporters. Eighteen transporter types have been annotated and compared. Column dendrograms show similarity based on Euclidean distance and hierarchical clustering. Gene clusters were determined by k-means clustering with Euclidean distance. The heatmap colour gradient shows low gene expression (blue) and high gene expression (red). **b** Expression trend of the stringent response signal ppGpp encoding gene *relA*. *relA* was identified as a significantly differentially expressed gene between these two treatments ($p < 0.05$, right-tailed Fisher's exact *t* test followed by Benjamin-Hochberg (BH) adjustment). Data (TPM) are presented as box plots (lower bound at 25th percentile, centre line at the median, upper bound at 75th percentile) with whiskers at minimum and maximum values. Statistical significance of *relA* was calculated from pairwise TPM comparisons of triplicate samples. "NaCl" indicates the supplementation of additional NaCl (18 g/L) to $BG11_0$, while "None" indicates that no additional substances were added to $BG11_0$. Source data are provided as a Source Data file.

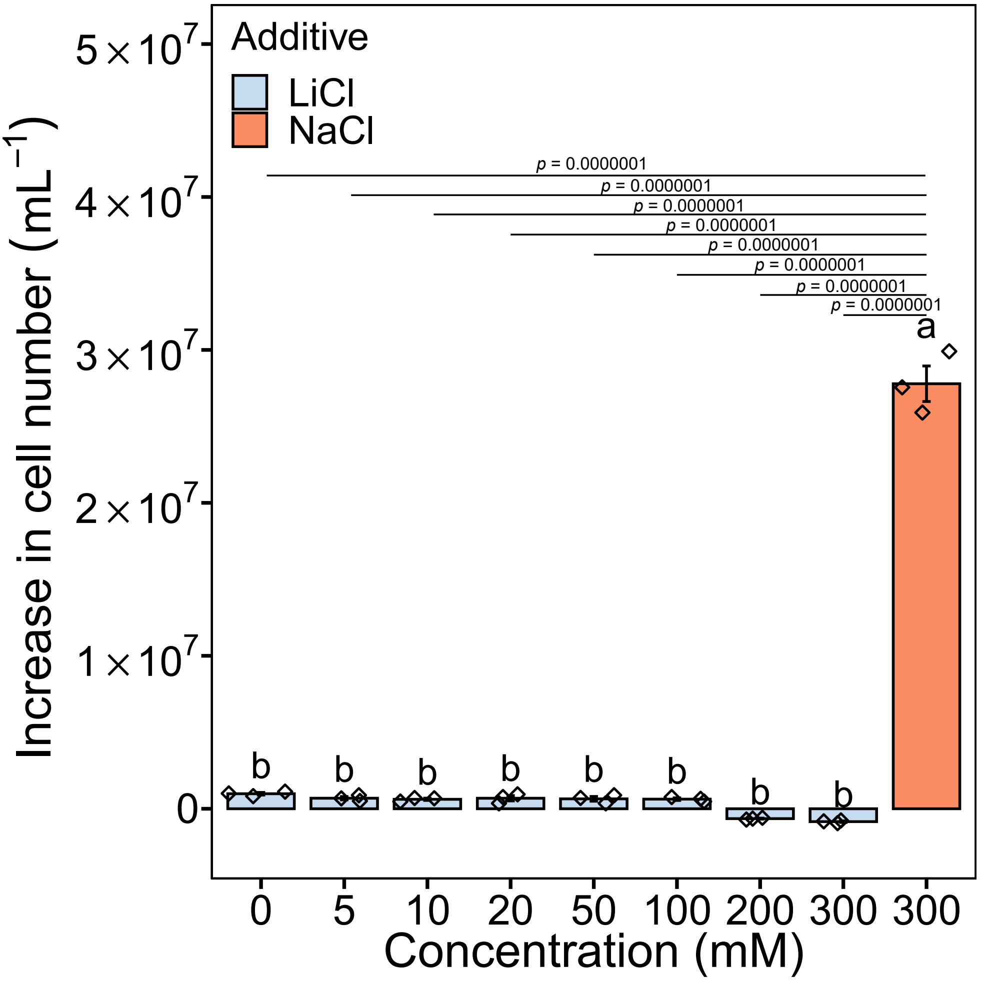

Supplementary Fig. 5. Population growth over seven days of cells grown in $BG11_0$ with LiCl. The increase in cell number of cells grown in $BG11_0$ with the addition of 0, 5, 10, 20, 50, 100, 200, 300 mM LiCl and 300 mM NaCl was calculated. The graph shows the mean ± standard deviation (n = 3 biologically independent samples). Different letters on each bar indicate statistical significance ($p < 0.05$) calculated by one-way ANOVA with Tukey's HSD post-hoc analysis over all populations tested. Source data are provided as a Source Data file.

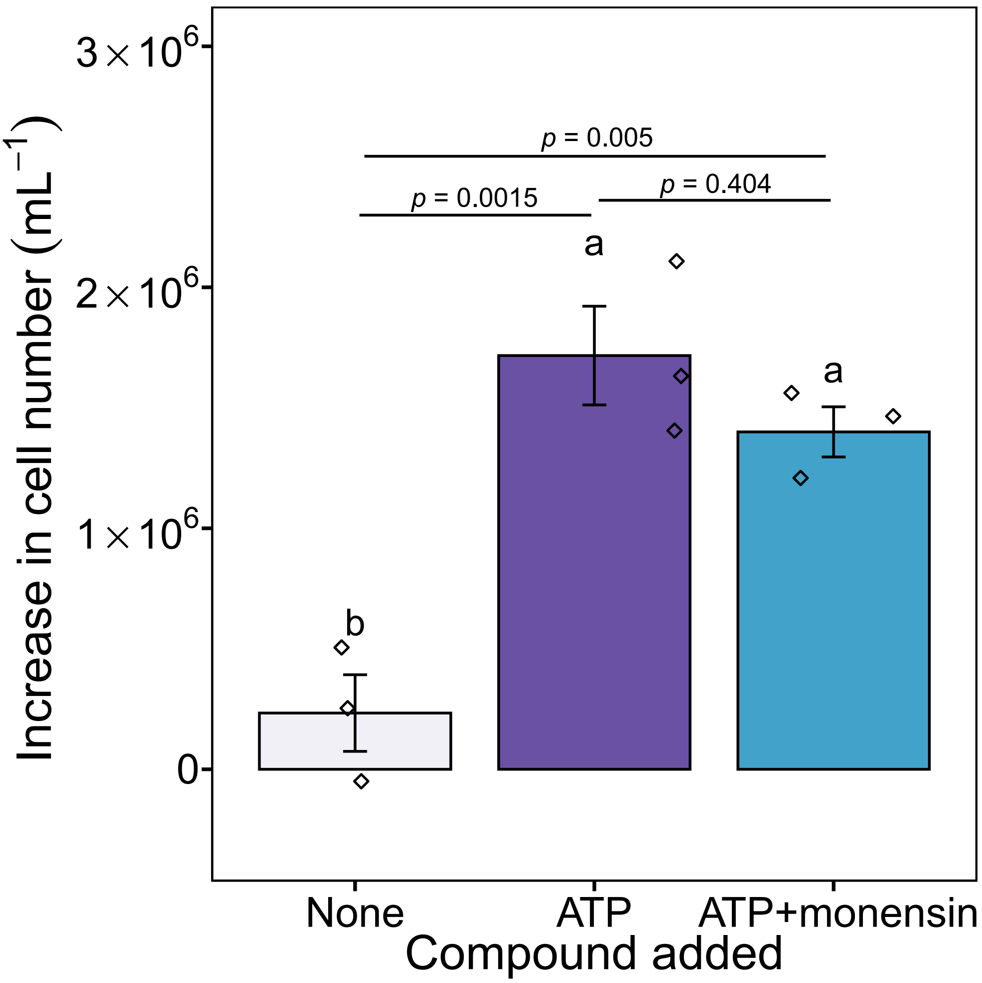

Supplementary Fig. 6. Population growth with ATP supplementation in the absence or presence of monensin over three days. Increase in cell number (3 days after inoculation) of cells grown in $BG11_0$, $BG11_0$ supplemented with 0.1 mM ATP or $BG11_0$ supplemented with 0.1 mM ATP and 14 μM monensin. The graph shows the mean ± standard deviation (n = 3 biologically independent samples). Different letters on each bar indicate statistical significance ($p < 0.05$) calculated by ANOVA with Tukey's HSD post-hoc analysis across all populations tested. Source data are provided as a Source Data file.

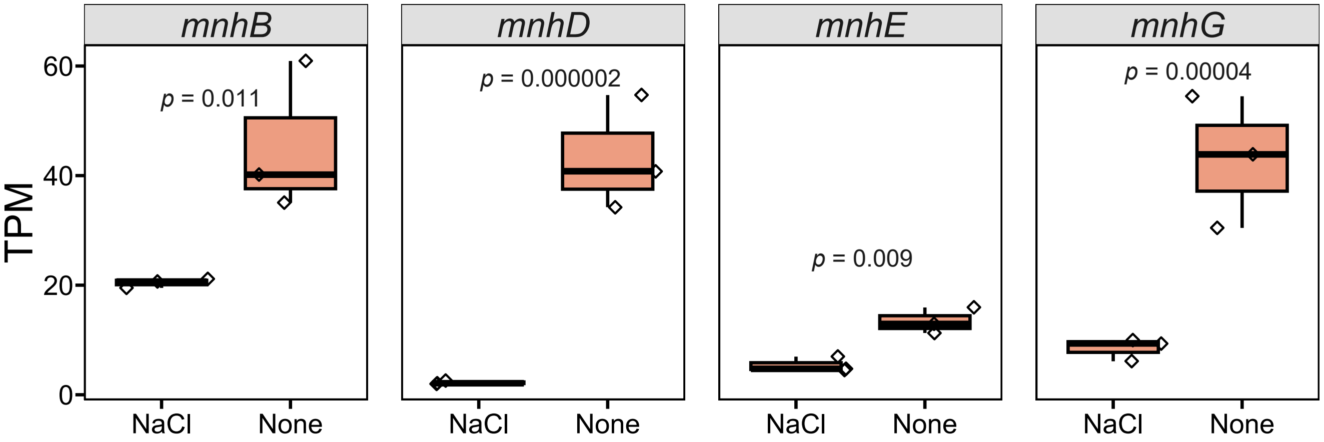

Supplementary Fig. 7. Expression trend of Na^+/H^+ antiporter encoding genes. All four Na^+/H^+ antiporter subunit encoding genes (subunit B (mnhB, K05566, KEGG), subunit D (mnhD, K05568, KEGG), subunit E (mnhE, K05569, KEGG), subunit G (mnhG, K05571, KEGG)) were identified as significantly differentially expressed genes between these two treatments ($p < 0.05$, right-tailed Fisher's exact *t* test followed by Benjamin-Hochberg (BH) adjustment). Data (TPM) are presented as box plots (lower bound at 25th percentile, centre line at the median, upper bound at 75th percentile) with whiskers at minimum and maximum values. Statistical significance of four genes was calculated from pairwise TPM comparisons of triplicate samples (grown in $BG11_0$) of NaCl deprivation treatment (None) compared to the NaCl supplementation (18 g/L) treatment (NaCl). Source data are provided as a Source Data file.

a

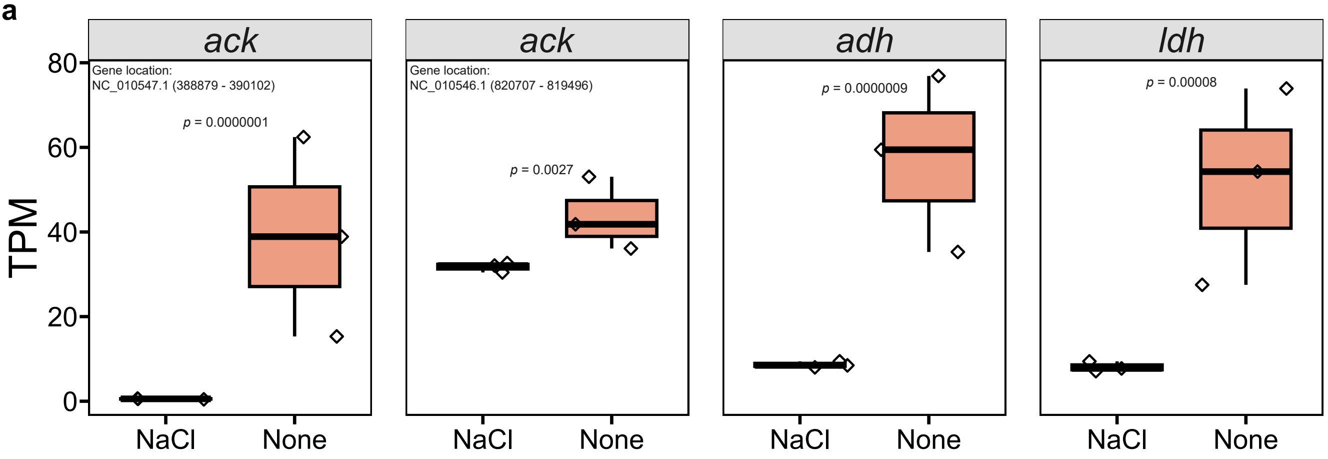
ack
ack
adh
ldh
TPM
80
60
40
20
0
Gene location:
NC_010547.1 (388879 - 390102)
p = 0.0000001
Gene location:
NC_010546.1 (820707 - 819496)
p = 0.0027
p = 0.0000009
p = 0.00008
NaCl
None
NaCl
None
NaCl
None
NaCl
None

b

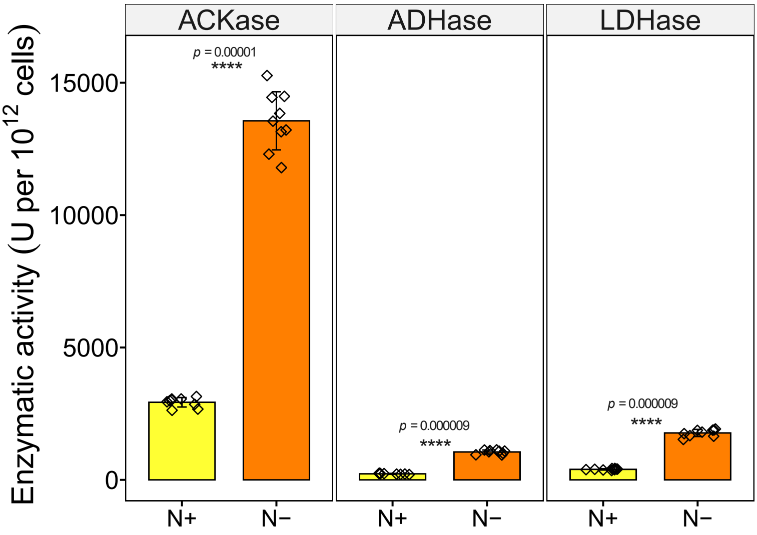
ACKase
ADHase
LDHase
Enzymatic activity (U per 10^12 cells)
15000
10000
5000
0
p = 0.00001
****
p = 0.000009
****
p = 0.000009
****
N+
N−
N+
N−
N+
N−

Supplementary Fig. 8. Transcript analysis of genes involved in fermentation and enzymatic activities of enzymes. a *ack* encoding acetate kinase (K00925, KEGG) (two ack genes were annotated at different locations of the genome), *adh* encoding alcohol dehydrogenase (K00001, KEGG), *ldh* encoding lactate dehydrogenase (K00016, KEGG). The locations of two *ack* genes are NC_010547.1 (388879 - 390102) and NC_010546.1 (820707 - 819496) in the NCBI database. Data (TPM) are presented as box plots (lower bound at 25th percentile, centre line at the median, upper bound at 75th percentile) with whiskers at minimum and maximum values. All three genes were identified as significantly differentially expressed genes between these two treatments ($p < 0.05$, right-tailed Fisher's exact *t* test followed by Benjamin-Hochberg (BH) adjustment). Statistical significance of genes analysed was calculated from pairwise TPM comparisons of triplicate samples of NaCl deprivation treatment (None) compared to the NaCl addition treatment (NaCl). **b** Enzymatic activities of ACKase, ADHase and LDHase. "N+" indicates BG11 with NaCl (18 g/L) while "N-" denotes $BG11_0$ with NaCl (18 g/L). The graphs show the mean ± standard deviation (n = 9 biologically independent samples). Corresponding significance markers represent statistical significance calculated by two-tailed Welch's *t* test. Significance: ns (no significance), *($p < 0.05$), **($p < 0.01$), ***($p < 0.001$), ****($p < 0.0001$). Source data are provided as a Source Data file.
