# Supplementary material for: A unicellular cyanobacterium relies on sodium energetics to fix N2
Source: Nat Commun. 2024 Nov 9;15:9716. doi: 10.1038/s41467-024-53978-9 (PMC11550448; doi:10.1038/s41467-024-53978-9)
Supplement: Supplementary file 2 — Reporting Summary [file 41467_2024_53978_MOESM2_ESM.pdf]

nature portfolio

Corresponding author(s): Katrin Hammerschmidt & Zhonghua Cai

Last updated by author(s): Oct 16, 2024

Reporting Summary

Nature Portfolio wishes to improve the reproducibility of the work that we publish. This form provides structure for consistency and transparency in reporting. For further information on Nature Portfolio policies, see our Editorial Policies and the Editorial Policy Checklist.

Statistics

For all statistical analyses, confirm that the following items are present in the figure legend, table legend, main text, or Methods section.

n/a	Confirmed	
☐	☒	The exact sample size (*n*) for each experimental group/condition, given as a discrete number and unit of measurement
☐	☒	A statement on whether measurements were taken from distinct samples or whether the same sample was measured repeatedly
☐	☒	The statistical test(s) used AND whether they are one- or two-sided<br>*Only common tests should be described solely by name; describe more complex techniques in the Methods section.*
☒	☐	A description of all covariates tested
☒	☐	A description of any assumptions or corrections, such as tests of normality and adjustment for multiple comparisons
☐	☒	A full description of the statistical parameters including central tendency (e.g. means) or other basic estimates (e.g. regression coefficient) AND variation (e.g. standard deviation) or associated estimates of uncertainty (e.g. confidence intervals)
☐	☒	For null hypothesis testing, the test statistic (e.g. *F*, *t*, *r*) with confidence intervals, effect sizes, degrees of freedom and *P* value noted<br>*Give P values as exact values whenever suitable.*
☒	☐	For Bayesian analysis, information on the choice of priors and Markov chain Monte Carlo settings
☒	☐	For hierarchical and complex designs, identification of the appropriate level for tests and full reporting of outcomes
☒	☐	Estimates of effect sizes (e.g. Cohen's *d*, Pearson's *r*), indicating how they were calculated

Our web collection on statistics for biologists contains articles on many of the points above.

Software and code

Policy information about availability of computer code

Data collection	RNA sequence data were analysed on the online platform Majorbio Cloud. Schematic illustrations were created by BioRender. R v4.1.1 was used to collect data in this study. R v4.1.1. was used for data visualisation. All scripts are available at https://github.com/SiTANG1990/N2-fixation-coastal-unicellular-cyanobacteria.git.
Data analysis	All data analyses were performed in R v4.1.1. Statistical significance for physiological measurements and fermentation-involved enzyme activity test was calculated by Welch's t test for pairwise comparisons of two treatments (p value < 0.05). A one-way ANOVA with Tukey's HSD post-hoc analysis (p value < 0.05) was conducted for all other assays, except for the Monension and DTHB (three-factor ANOVA) and DCCD test (two-way ANOVA). Differential gene expression (DGE) was conducted with DESeq2 v1.32.0. Heatmaps for transcriptomic data visualisation were generated using ComplexHeatmap v2.8.0.

For manuscripts utilizing custom algorithms or software that are central to the research but not yet described in published literature, software must be made available to editors and reviewers. We strongly encourage code deposition in a community repository (e.g. GitHub). See the Nature Portfolio guidelines for submitting code & software for further information.

Data

Policy information about availability of data

All manuscripts must include a data availability statement. This statement should provide the following information, where applicable:

- Accession codes, unique identifiers, or web links for publicly available datasets
- A description of any restrictions on data availability
- For clinical datasets or third party data, please ensure that the statement adheres to our policy

The data of this study are available within the article. Raw RNAseq reads for differential gene expression analyses have been submitted to NCBI's SRA database (http://www.ncbi.nlm.nih.gov) under BioProject PRJNA1014498. The KEGG database (https://www.kegg.jp/) was used for functional enrichment analyses. Source data are provided with this paper.

Research involving human participants, their data, or biological material

Policy information about studies with human participants or human data. See also policy information about sex, gender (identity/presentation), and sexual orientation and race, ethnicity and racism.

Reporting on sex and gender	There is no such information in this study.
Reporting on race, ethnicity, or other socially relevant groupings	There is no such information in this study.
Population characteristics	There is no such information in this study.
Recruitment	There is no such information in this study.
Ethics oversight	There is no such information in this study.

Note that full information on the approval of the study protocol must also be provided in the manuscript.

Field-specific reporting

Please select the one below that is the best fit for your research. If you are not sure, read the appropriate sections before making your selection.

☐ Life sciences ☐ Behavioural & social sciences ☒ Ecological, evolutionary & environmental sciences

For a reference copy of the document with all sections, see nature.com/documents/nr-reporting-summary-flat.pdf

Ecological, evolutionary & environmental sciences study design

All studies must disclose on these points even when the disclosure is negative.

Study description	In this study, we tested the effect of NaCl on the N2 fixation of a unicellular coastal cyanobacteria Cyanothece sp. ATCC 51142, together with growth experiments, enzyme quantification, enzyme activity estimation, quantification of physiological parameters, transcriptomics. In these quantification experiments (factorial design), all treatments, e.g., cells grown in N-deficient medium with or without NaCl, had three replicates (enzyme activity estimation tests had 6 to 9 replicates), and for transcriptomic analysis, triplicate randomly-sampled cultures of two treatments were investigated. For other experiments, exact n values were provided in the manuscript. Samples were harvested to have enough representation from each treatment.
Research sample	The research sample studied in this study was the well-known unicellular N2 fixation cyanobacterium, Cyanothece sp. ATCC 51142. It was purchased from the American Type Culture Collection (ATCC), USA. The strain was chosen as the model organism due to the observation of its NaCl-dependent N2 fixation, which conflicts with the traditional view that coastal N2 fixation was inhibited by NaCl. During the experiments, cells were grown in N-deficient medium with or without NaCl and were collected for further analyses.
Sampling strategy	No statistical methods were used to predetermine sample size in this study. In general, n = 3 was chosen as the replicate number based on previous experience and standards in the field (https://doi.org/10.1038/s41396-022-01307-7). For enzyme activity quantification, n = 6 or 9 was measured for each treatment.
Data collection	Transcriptomics was performed by Shanghai Majorbio Bio-pharm Technology Co., Ltd. (Shanghai, China). Protein sequence alignment analysis was conducted by Si Tang using the Jalview software. Yaqing Liu recorded the data from the quantification of physiological parameters, and Si Tang, Xueyu Cheng recorded the data from all other experiments, these data were collected and recorded manually with pen and paper.
Timing and spatial scale	We first conducted several rounds of pre-experiments, and based on the pre-results, we designed the main experiments. A series of main experiments were conducted from November 2022 to November 2023. Transcriptomics was conduted in June, 2023. The LiCl experiment, glycogen quantification, amiloride test and cellular ATP, ATP/ADP ratio analyses were conducted in June to July, 2024. There was no gap between collection periods and no spatial scale from which the data were taken.

Data exclusions	No data were excluded from the analysis.
Reproducibility	In general, all experiments were done in triplicate, and all attempts of replication were successful and showed similar results. For our central observation (NaCl-dependent N2 fixation), we conducted four pre-experiments before collecting formal data, and all trials resulted in similar results. For physiological data, their patterns were consistent with previously known results.
Randomization	Since our model organism is photoautotrophic, during experiments, the positions of culture flasks were randomly mixed daily to reduce possible differences in growth due to light availability. Cyanobacterial cell cultures were gently mixed for randomization for all experiments in this study before pipetting out for further analysis.
Blinding	The persons analyzing data were unaware of the sample identity and group allocation. Numerical data have been analyzed double-masked, resulting in the same results as in the non-blinded analysis.

Did the study involve field work? ☐ Yes ☒ No

Reporting for specific materials, systems and methods

We require information from authors about some types of materials, experimental systems and methods used in many studies. Here, indicate whether each material, system or method listed is relevant to your study. If you are not sure if a list item applies to your research, read the appropriate section before selecting a response.

Materials & experimental systems

n/a	Involved in the study
☒	☐ Antibodies
☒	☐ Eukaryotic cell lines
☒	☐ Palaeontology and archaeology
☒	☐ Animals and other organisms
☒	☐ Clinical data
☒	☐ Dual use research of concern
☒	☐ Plants

Methods

n/a	Involved in the study
☒	☐ ChIP-seq
☒	☐ Flow cytometry
☒	☐ MRI-based neuroimaging

Plants

Seed stocks	There is no such information in this study.
Novel plant genotypes	There is no such information in this study.
Authentication	There is no such information in this study.
